# Preclinical evaluation of a fully threaded biodegradable magnesium alloy headless compression screw for enhanced fixation in small bones

**DOI:** 10.1016/j.jot.2026.101201

**Published:** 2026-08-27

**Authors:** Hiroshi Noguchi, Kotaro Hanada, Ryunosuke Watanabe, Yasukazu Totoki, Yohei Tomaru, Akira Ikumi, Akira Kitagawa, Sakiko Orui, Lulu Zhang, Hajime Mishima

**Affiliations:** a Department of Orthopaedic Surgery, Institute of Medicine, University of Tsukuba, Ibaraki, 305-8575, Japan; b Integrated Research Center for Advanced Manufacturing, National Institute of Advanced Industrial Science and Technology (AIST), Ibaraki, 305-8564, Japan; c Mel-frontier Ltd., Tokyo, 170-0013, Japan; d National Metrology Institute of Japan (NMIJ), National Institute of Advanced Industrial Science and Technology (AIST), Ibaraki, 305-8565, Japan

**Keywords:** Biodegradable magnesium alloy, Biological safety, Fully threaded compression screw, Histological evaluation, In vivo, Mechanical performance

## Abstract

**Background:**

Biodegradable magnesium (Mg) alloys have attracted attention as alternatives to conventional metallic implants; however, their clinical applicability to fully threaded headless compression (HC) screws has been scarcely investigated.

**Methods:**

In this study, a fully threaded biodegradable Mg alloy HC screw was developed, and its mechanical performance, biological safety, and in vivo degradation behavior were systematically evaluated. Mechanical properties were assessed according to ASTM standards and compared with clinically used titanium (Ti) alloy and poly-L-lactic acid/hydroxyapatite (PLLA/HA) screws. Biological safety was evaluated in accordance with ISO 10993 and in compliance with Good Laboratory Practice. In vivo degradation behavior was investigated using a rabbit distal femoral condyle model.

**Results:**

The Mg alloy HC screw demonstrated superior torsional strength compared with PLLA/HA screws and comparable insertion performance, along with enhanced fixation strength, compared with Ti alloy screws. No cytotoxicity, sensitization, or intracutaneous reactivity was observed. Although transient gas cavity formation was observed during the early implantation period, it progressively diminished over time and did not adversely affect bone formation or implant stability.

**Conclusion:**

These findings indicate that the developed Mg alloy HC screw exhibits favorable mechanical performance, biocompatibility, and in vivo degradation. This study provides preclinical evidence supporting its potential clinical translation, particularly in eliminating the need for secondary implant removal surgery.

**The translational potential of this article:**

This article focuses on the preclinical evaluation of a fully threaded Mg-alloy HC screw developed to improve fixation performance for small bone fractures. The results of this study have significant clinical potential for the fully threaded design of biodegradable Mg alloy implants in fracture treatment.

## Graphical abstract

**Figure:**
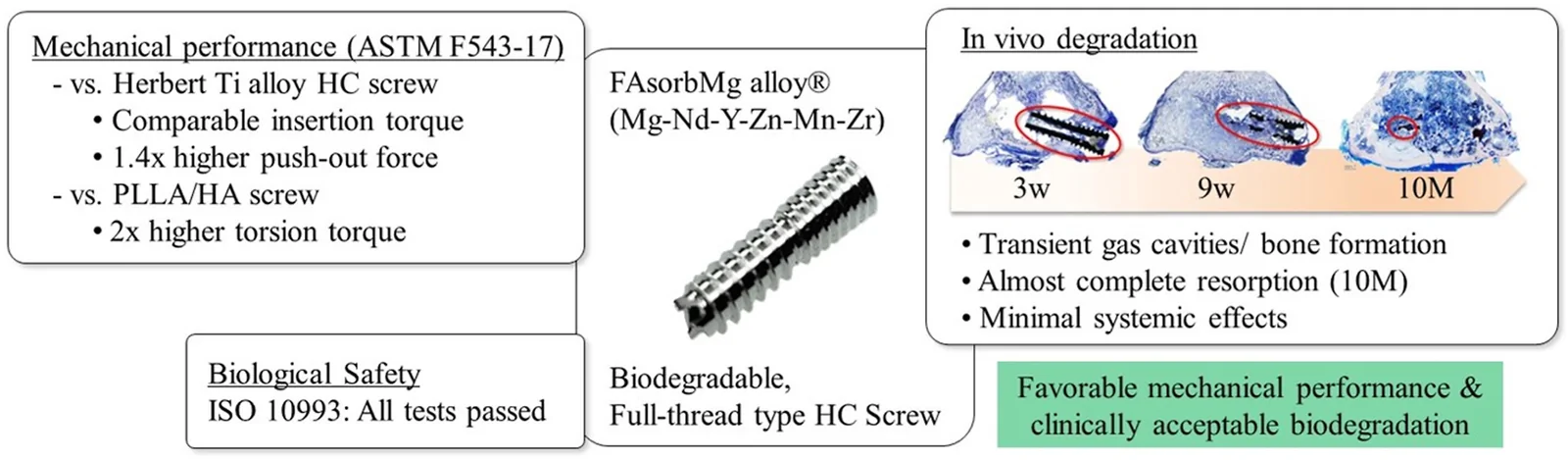

## 1 Introduction

Headless compression (HC) screws used in orthopedic treatment of bone fractures are internal fixation implants with a cannulated structure for guidewire insertion and threads of different pitches at the leading and trailing ends. This design enables precise fixation of the fracture site and promotes bone healing through the compressive force generated by the pitch differential during insertion. Compared with headed screws, such as conventional cortical bone screws, HC screws can be completely countersunk within the bone, thereby reducing soft-tissue irritation and damage at the implantation site. Consequently, HC screws are preferred for internal fixation in procedures such as carpal bone fractures and small-fragment fractures. Currently, titanium (Ti) alloys and stainless steel, i.e., non-degradable metallic materials, are considered the gold standard for HC screws due to their high mechanical strength and excellent biocompatibility [1]. However, these non-degradable screws can lead to clinical issues when implanted long-term in the body, including inhibition of skeletal growth (particularly in pediatric patients), irritation-induced rupture or inflammation of the surrounding tissues due to implant prominence, and imaging artifacts during radiographic examinations [[2], [3], [4]]. As a result, secondary removal surgery may be required after fracture healing, imposing a significant burden on patients.

The application of biodegradable materials to HC screws, which are gradually degraded and resorbed and eventually replaced by biological tissue, is expected to address the above-mentioned clinical issues caused by long-term implantation and to eliminate the need for secondary removal surgery. Representative biodegradable materials include biodegradable polymers such as poly-L-lactic acid (PLLA) and polyglycolic acid, as well as biodegradable metals, such as magnesium (Mg) alloys. Although biodegradable polymers exhibit relatively stable degradation behavior, their markedly low mechanical strength leads to a high risk of screw fracture and insufficient fixation strength at the fracture site [5,6]. In contrast, Mg alloys have attracted considerable attention as promising candidates for HC screws because they have mechanical properties close to those of bone, excellent biocompatibility, and osteoconductive and osteogenic potential [[7], [8], [9], [10], [11]].

Numerous studies, particularly in Europe, have investigated Herbert-type MgYREZr (WE43) alloy HC screws with a double-thread design. These studies have demonstrated that such screws exhibit safety and therapeutic efficacy comparable to those of Ti alloy HC screws with the same design, suggesting their potential as a favorable fixation option that may prevent secondary screw removal surgery [[12], [13], [14], [15], [16], [17], [18], [19], [20], [21], [22], [23], [24], [25], [26], [27], [28]]. However, Herbert-type Mg alloy screws, which maintain moderate compressive force through threads at the leading and trailing ends, are at risk of decreasing compressive and holding force due to the thread degradation and gas cavity formation in the body. Further improving their mechanical performance remains a significant challenge. Full-thread-type HC screws, which have threads along the entire screw length, offer advantages over Herbert-type screws, including stronger and wider compressive and holding forces at the fracture site, as demonstrated by mechanical evaluations [[29], [30], [31], [32]]. However, the use of Mg alloys in fully threaded HC screws has not been thoroughly investigated to date, particularly with respect to their mechanical reliability, in vivo degradation behavior, and biological safety. Therefore, this study aimed to develop a fully threaded Mg alloy HC screw and to comprehensively evaluate these properties from a translational perspective.

## 2 Materials and methods

### 2.1 Material preparation and design of Mg alloy HC screw

FAsorbMg alloy® (Mg-2.5Nd-1Y-0.5Zn-0.1Mn-0.1Zr, wt%; Fuji Light Metal Co., Ltd.), which exhibits excellent mechanical properties and corrosion resistance (ultimate tensile strength: 222 MPa, elongation at break: 35%, corrosion rate: 0.1 mm/year), was used as the base material for the Mg alloy HC screws. This alloy has also been used in other biodegradable medical devices, such as surgical staples [33]. The alloy billet was hot-extruded into a 6 mm diameter rod at an extrusion ratio of 20 and a processing temperature of 400 °C, followed by fabrication into HC screws using CNC lathe machining. As shown in Fig. 1, the fabricated Mg alloy HC screw features a full-thread design with an overall length of 10 mm. The geometrical specifications include a 6-mm-long straight section at the leading end (outer diameter: 2.8 mm, pitch: 0.75 mm, thread height: 0.4 mm) and a 4-mm-long tapered section at the trailing end (outer diameter: 3.2 mm, pitch: 0.55 mm, taper angle: 5°). All fabricated HC screws were heat-treated and chemically converted before being provided for each assessment.

**Fig. 1:**
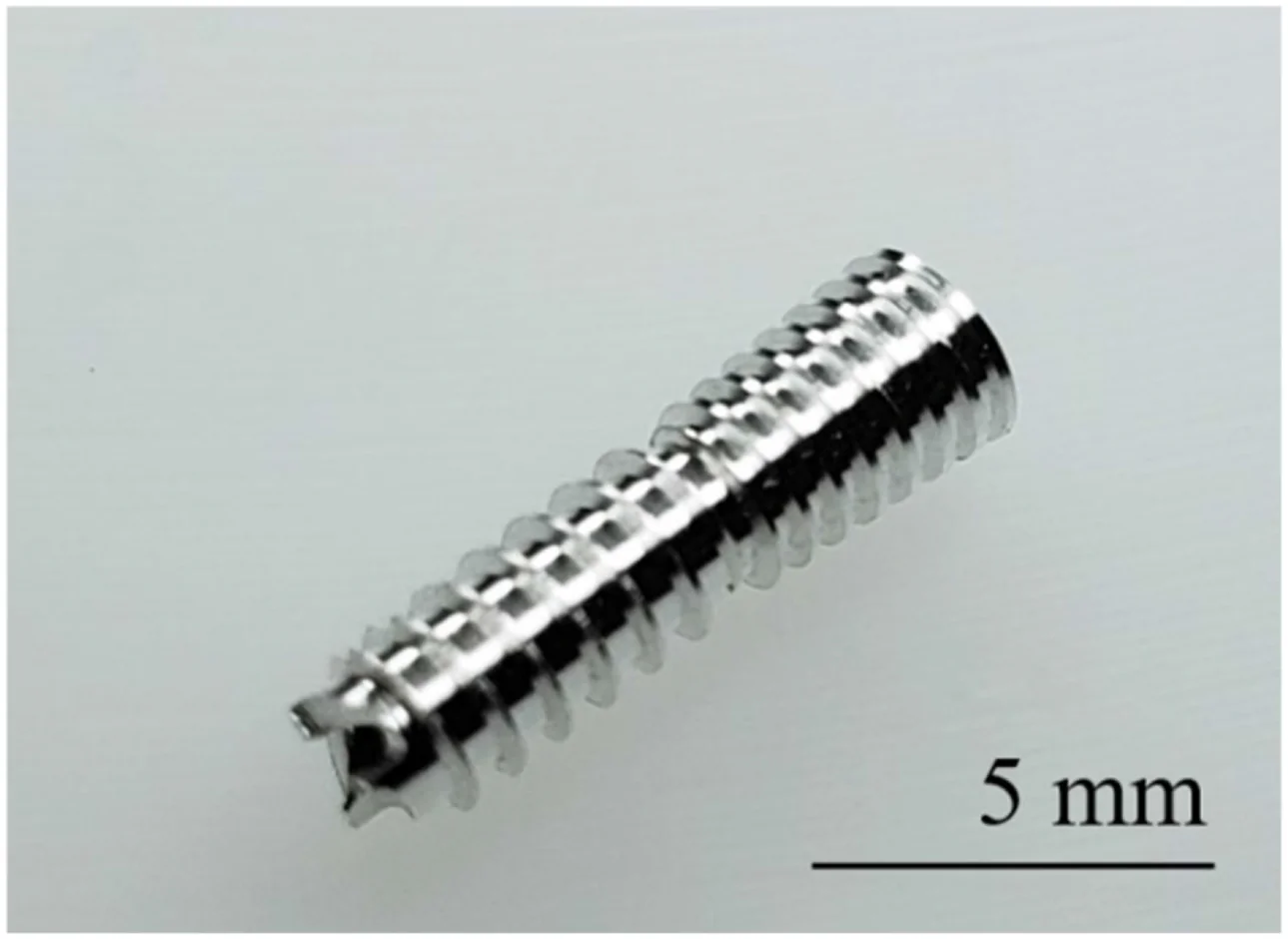
Appearance of full-thread type Mg alloy HC screw.

### 2.2 Initial mechanical safety assessment

#### 2.2.1 Screw samples

To assess the initial mechanical performance of the fabricated Mg alloy HC screws, a series of mechanical tests was conducted in accordance with ASTM F543-17, including torsional, driving torque, and push-out tests. Solid rigid polyurethane (SRP) foam block (20 pcf, Sawbones) was used as an alternative testing material for human cancellous bone in accordance with ASTM F1839-08. A Herbert-type double-threaded Ti alloy screw (CS 3.0, DePuy Synthes) and a non-headless PLLA/hydroxyapatite (HA) screw (SuperFIXSORB 30, Teijin Medical Technologies) were selected as reference samples for mechanical testing. These screws represent typical clinical standards for non-degradable metallic and biodegradable fixation devices, respectively, and served as benchmarks for mechanical performance. Table 1 summarizes the screws used in this safety assessment.

**Table 1:** Specifications of the compression screws used in the mechanical safety assessment.

Product	Material	Specification	
Mg alloy screw	FAsorbMg®, Degradable	Canulated, Headless and Full-thread type, O.D. 2.6-3.0 mm, I.D. 1.0 mm, L10 mm	
CS 3.0	Ti6Al4V alloy, Non-degradable	Canulated, Headless and double-thread (Harbert) type, O.D. 3.0-2.0-3.5 mm, I.D. 1.2 mm, L12 mm	
SuperFIXSORB 30	70%PLLA /30%HA, Degradable	Solid, Cortical screw type, O.D. 2.7 mm, L12 mm	

#### 2.2.2 Insertion torque test

To evaluate screw insertability, insertion torque was measured using a torsion testing machine (Resolution: 1.13 × 10^−5^ Nm, MT1, Instron). A 40 mm × 40 mm SRP foam block with a pre-drilled through-hole for screw insertion was mounted on the torsion testing machine. Using a screwdriver, a constant axial load of 10 N was applied to the screw, which was rotated clockwise at 1 rpm. Torque and angular displacement were measured until the screw head was fully countersunk. For the PLLA/HA screws, which lack a self-tapping function like the Mg alloy and Ti alloy screws, the insertion test was conducted after tapping the through-hole.

#### 2.2.3 Torsion test

The torsional properties of the screws were also measured using the same torsion testing machine (Resolution: 1.13 × 10^−5^ Nm, MT1, Instron) as the insertion torque test. A stainless-steel guide wire was inserted into each screw, and the screw was held in the machine's chuck, leaving five threads from the screw end. Using a screwdriver, a constant axial load of 10 N was applied to the screw, and the screw was rotated clockwise at 1 rpm. Torsional torque and angular displacement were measured until the screw fractured.

#### 2.2.4 Push-out test

The pull-out properties of screws inserted into SRP foam blocks were measured using a push-out test on a universal testing machine (Autograph AG-100kN XPlus, Shimadzu). For the screws inserted into the SRP foam blocks in the insertion torque test, a punch was placed at the screw tip and pushed axially at a crosshead speed of 10 mm/min. Load and displacement were measured during the test.

### 2.3 Biological safety assessment

The biological safety of the FAsorbMg alloy was evaluated for cytotoxicity, sensitization, and intracutaneous reactivity using cell culture assays and animal studies in accordance with ISO 10993 and Good Laboratory Practice (GLP).

#### 2.3.1 Cytotoxicity

Cytotoxicity testing of the Mg alloy was conducted using alloy extracts, together with negative and positive controls. A stock Mg alloy extract was prepared by adding an extraction solvent (1 mL per 6 cm^2^ of surface area) to sterilized alloy samples, followed by incubation at 37 °C for 24 h under agitation. The stock extract was diluted to 10% (v/v) in Eagle's Minimum Essential Medium (EMEM) (Nissui Pharmaceutical) supplemented with 5% fetal bovine serum (FBS), and this solution was defined as the highest test extract concentration. This test extract was further diluted with EMEM to obtain final concentrations of 3.13%, 6.25%, 12.5%, 25%, 50%, and 80%. A blank control (0% test extract) was also prepared using the same extraction procedure without Mg alloy samples. As a positive control, dimethyl sulfoxide solutions containing zinc dibutyldithiocarbamate were prepared in EMEM at concentrations of 0, 1, 2, 3, and 4 μg/mL. Chinese hamster lung fibroblasts (JCRB0603: V79) were suspended in EMEM and seeded into multi-well plates at a density of 100 cells per well in 2 mL of medium, then cultured for 24 h at 37 °C in a humidified atmosphere containing 5% CO_2_. The culture medium was then replaced with the respective test or control solutions, and the cells were cultured for an additional 6 days. After incubation, the cells were washed with phosphate-buffered saline, fixed with methanol, and stained with Giemsa. Colonies consisting of 50 or more cells in each well were counted under a stereomicroscope (SZ61TRC-C-D, Olympus). Cell viability was calculated relative to the mean colony count of the blank control. A sample was considered cytotoxic if its cell viability was less than 70%.

#### 2.3.2 Skin sensitization

The skin sensitization potential of the Mg alloy was evaluated using the guinea pig maximization test (GPMT). Sterilized Mg alloy samples were extracted in either polar or non-polar extraction solvents (1 mL per 3 cm^2^ of surface area) at 37 °C for 72 h under gentle agitation. For the intradermal induction phase, 0.1 mL of the obtained extracts was intradermally injected into the scapular region of female Hartley guinea pigs (5 weeks old; Japan SLC; n = 10). 8 days later, a patch application was performed for the topical induction phase. Subsequently, 14 days after the topical induction, a 24-h topical patch was applied to the abdominal region for the challenge phase. After the challenge, skin reactions were observed at 24 and 48 h after patch removal and graded on a scale from 0 to 3 according to the Magnusson and Kligman grading scale. Mean grades were calculated for each group. The same evaluation was conducted for the blank control group (extraction solvent incubated without samples) and the positive control group (0.1% 1-chloro-2,4-dinitrobenzene (DNCB) in a non-polar solvent), each consisting of five animals. Skin sensitization was considered positive if the mean grade was ≥1.0.

#### 2.3.3 Intracutaneous reactivity

An intracutaneous reactivity test was conducted to evaluate the skin tissue response to Mg alloy extracts. Sterilized Mg alloy samples were immersed in either polar or non-polar extraction solvents (1 mL per 3 cm^2^ of surface area) and extracted at 37 °C for 72 h under gentle agitation. The obtained extracts, along with corresponding blank extracts, were intradermally injected (0.2 mL per site) into 5 sites per side of the dorsal skin (10 sites in total) of male Japanese white rabbits (19 weeks old; Japan SLC; n = 3). Skin reactions (erythema and edema) were observed macroscopically at 24, 48, and 72 h post-injection and scored from 0 to 4 according to reaction severity (0: no change, 1: minimal, 2: mild, 3: moderate, 4: marked). Irritation was considered negative if the difference between the mean reaction score of the extract and that of the blank control was ≤1.0 and positive if the difference was > 1.0.

### 2.4 In vivo study

#### 2.4.1 Animal model and surgery

Female Japanese white rabbits (12 weeks old; approximately 2.5 kg body weight; Kitayama Labes; n = 10) were used to evaluate the time-dependent biodegradable behavior of the implanted Mg alloy HC screws. The rabbits were anesthetized via intramuscular injection of ketamine hydrochloride (Ketalar, 50 mg/mL; 1.0 mL/kg) and xylazine hydrochloride (Celactal, 20 mg/mL; 0.7 mL/kg). A longitudinal incision was made on the lateral side of the knee joint to expose the lateral femur, and a 0.8-mm-diameter guidewire was inserted from the lateral femoral epicondyle toward the medial side of the femur. Subsequently, a bone hole was prepared over the guidewire using a 2.1-mm-diameter cannulated straight drill, followed by a cannulated countersink. The Mg alloy HC screw was then inserted into the predrilled bone hole. The same procedure was performed on both femora of each rabbit. Femoral samples containing the implanted screws were harvested postoperatively at 3, 6, and 9 weeks for short-term assessment (n = 6 at each time point) and at 10 months for long-term assessment (n = 2). All animal experiments were conducted in accordance with relevant guidelines and regulations, with meticulous attention to animal health management and pain mitigation.

#### 2.4.2 Radiological evaluation

Radiography was performed with an X-ray imaging system (ADX4000V-L, OSADA ELECTRIC) on the harvested femoral samples to assess time-dependent changes in the implanted Mg alloy HC screws and surrounding bone tissue. In addition, femoral samples embedded in methyl methacrylate (MMA) resin were imaged using micro–X-ray computed tomography (micro-CT; SMX-160CT-SV3, Shimadzu) at 65 kV to reconstruct three-dimensional images of the screws and determine their volumes.

#### 2.4.3 Histological evaluation

The harvested femoral samples containing the implanted screw were fixed in 10% neutral buffered formalin and embedded in MMA resin. Approximately 30-μm-thick undecalcified ground sections were prepared from the femoral condyle along the long axis of the screw using a precision cutting system (BS-3000 N, EXAKT GmbH) and a grinding system (MG-4000 N, EXAKT GmbH). The sections were stained with Toluidine Blue (TB) for histological evaluation. All prepared sections were observed using an optical microscope (BZ-X710, KEYENCE). The area fractions of gas cavities and bone tissue around the screw relative to the total cross-sectional area of the femoral condyle were determined using ImageJ software (U.S. National Institutes of Health). In low-magnification images, inflammatory responses around the screw, bone/fibrous tissue-implant contact, and osteocyte necrosis were each scored on a scale from 0 to 5 according to the severity of the response (0: no change, 1: minimal, 2: mild, 3: moderate, 4: marked, 5: severe/extreme). In addition, high-magnification images were used to assess bone contact and formation at the screw surface qualitatively.

#### 2.4.4 Elemental analysis

To evaluate the degradation products of Mg alloy screws after long-term implantation, X-ray fluorescence (XRF) mapping was performed using an X-ray microscope (XGT-5000, HORIBA). The analysis was conducted on rabbit femoral samples harvested after 10 months of implantation, embedded in MMA resin, cut, and polished to #1000. Furthermore, inductively coupled plasma mass spectrometry (ICP-MS) (Limit of detection: pg/g, Agilent 8800, Agilent Technologies) was performed on rabbit organ samples harvested after implantation to determine the concentrations of metal elements accumulated in the organs as a result of screw degradation. Blood, liver, kidney, and spleen samples harvested from rabbits at 9 weeks and 10 months post-implantation were weighed into quartz beakers, subjected to thermal acid digestion with sulfuric and nitric acids, diluted with ultrapure water to a defined final volume, and then analyzed by ICP-MS.

## 3 Results and discussion

### 3.1 Initial mechanical performance

Fig. 2 shows the results of the initial mechanical safety assessment of the Mg alloy HC screw and commercially available, clinically approved screws. The insertion torque of the Mg alloy screw gradually increased as insertion progressed and further increased upon reaching the tapered tail section; however, no sudden torque increase was observed, in contrast to the Ti alloy and PLLA/HA screws. The maximum insertion torque of the Mg alloy screw was 0.15 Nm, which was comparable to that of the Ti alloy screw (0.18 Nm).

**Fig. 2:**
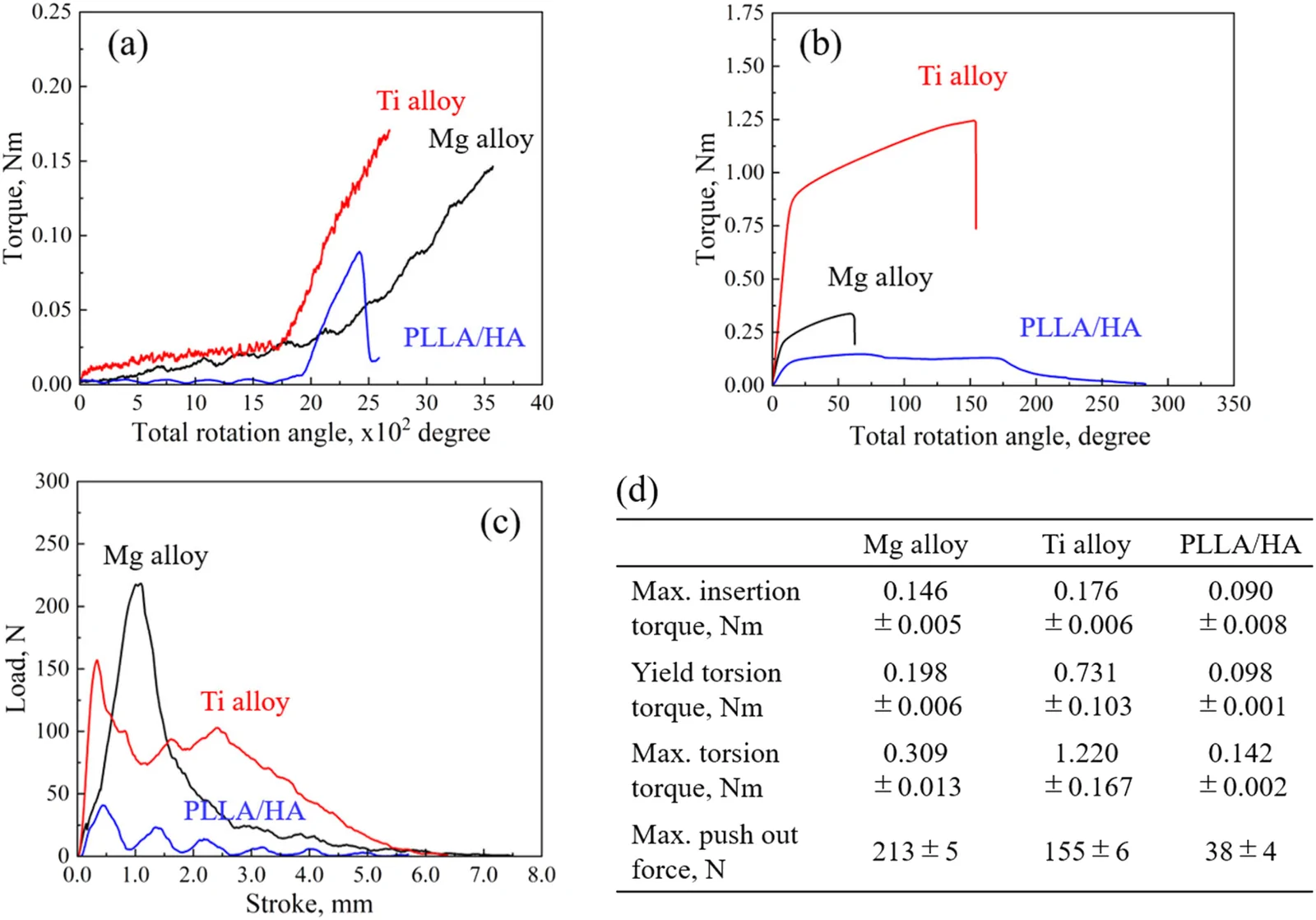
Initial mechanical safety assessment results for the developed Mg alloy HC screw and commercially available screws: (a) insertion torque curve, (b) torsion torque curve, (c) push-out force curve, and (d) screw properties.

The yield torsion torque and maximum torsion torque of the Mg alloy screw were 0.20 Nm and 0.31 Nm, respectively, corresponding to approximately one-fourth those of the Ti alloy screw and approximately twice those of the biodegradable PLLA/HA screw.

The maximum push-out force for the full-thread-type Mg alloy screw in the SRP foam block was 213 N. This value was approximately 1.4 times higher than that of the Herbert-type Ti alloy screw and more than five times higher than that of the full-thread-type PLLA/HA screw.

These results from the initial mechanical safety assessment indicate that the developed Mg alloy HC screw exhibits significantly higher insertion and maximum torques than the clinically approved biodegradable PLLA/HA screw. In addition, its insertability, fixation, and pull-out resistance are comparable to or even superior to those of the Ti alloy screw. These findings indicate that the developed Mg alloy HC screw meets the mechanical requirements for clinical use.

### 3.2 Biological safety

Fig. 3 shows the cytotoxicity test results for the test extract prepared from the Mg alloy and the positive control. The test extract showed a cell viability of approximately 100% at all concentrations. In contrast, the positive control reduced cell viability to below 70% at concentrations of 3 μg/mL or higher. The IC50 (concentration resulting in 50% cell viability) determined via logarithmic regression was 3.01 μg/mL, meeting the acceptance criteria. These results indicate that the Mg alloy used for the screw components is classified as non-cytotoxic.

**Fig. 3:**
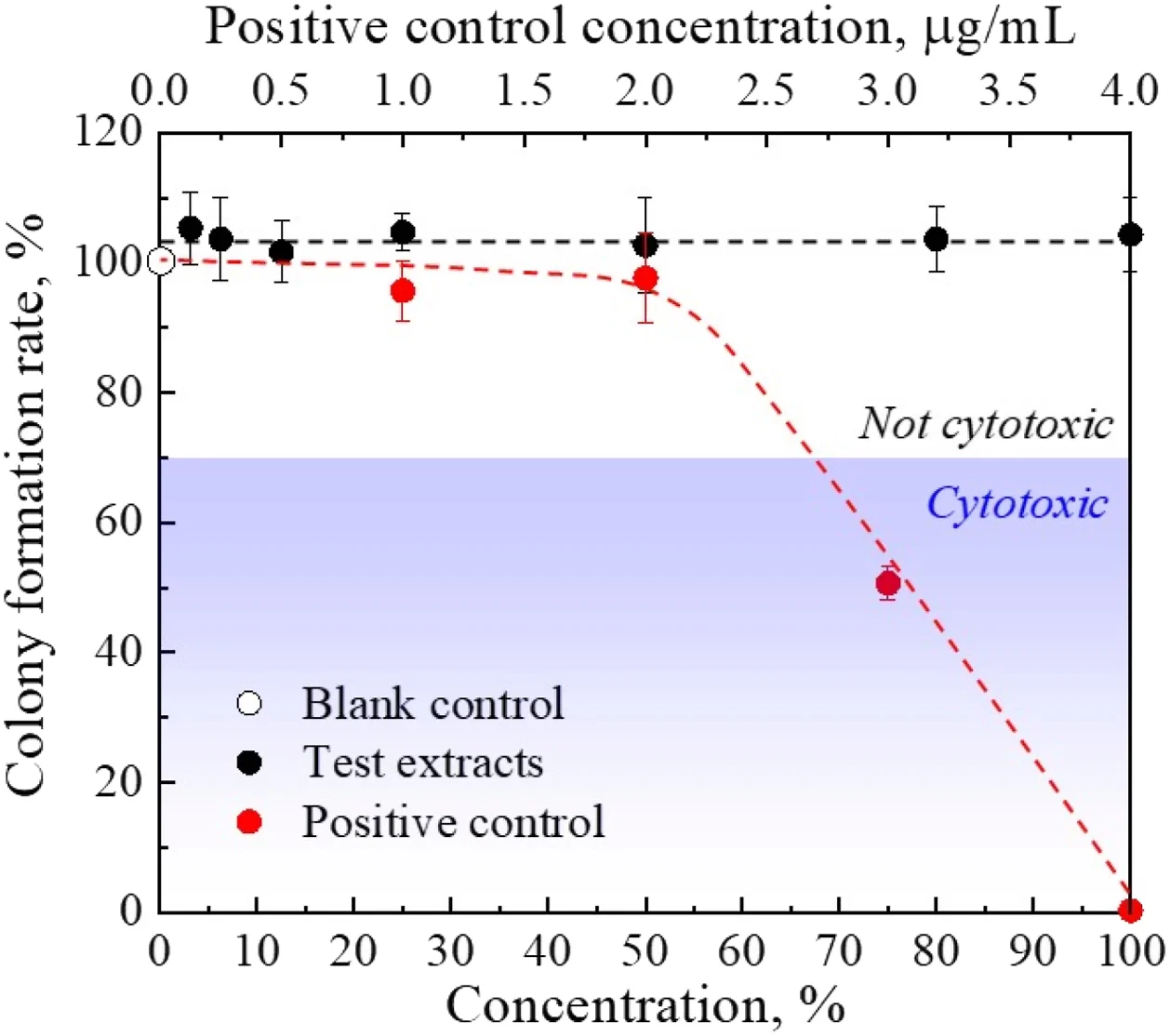
Cytotoxicity test results for the test extract prepared from the Mg alloy and the positive control.

Table 2 shows the GPMT results for the Mg alloy extracts. At 24 and 48 h after removal of the challenge patch, no abnormal skin reactions were observed for either the Mg alloy extracts or the blank controls, with a mean score of 0.0 (negative). In contrast, the positive control exhibited severe erythema and edema, with a mean score of 3.0 (positive). Based on these results, the Mg alloy was determined to be non-sensitizing under the test conditions.

**Table 2:** Skin sensitization results of Mg alloy extracts in GPMT.

Group	Test solvent	Mean score^a^		Sensitization potential
		24 h	48 h	
Blank control	Polar	0.0	0.0	Negative
	Non-polar	0.0	0.0	Negative
Mg alloy extract	Polar	0.0	0.0	Negative
	Non-polar	0.0	0.0	Negative
Positive control	Non-polar (0.1%DNCB)	3.0	3.0	Positive

a Grading of findings: no change 0, mild 1, moderate 2, marked 3.

Fig. 4 shows representative images of rabbits after the intracutaneous reactivity test with the Mg alloy extracts. For the polar extracts, no erythema or edema was observed at any injection site throughout the observation period, with a mean score of 0.0. For the polar blank controls, very mild erythema was observed at a single site at 24 h post-injection; however, no erythema or edema was observed at any other sites, resulting in a mean score of 0.03. The mean score difference between the polar extracts and polar blank controls was −0.03, indicating a negative intracutaneous reactivity response. For the non-polar extracts and non-polar blank controls, no erythema or edema was observed at any injection site throughout the observation period, with a mean score of 0.0. Consequently, the mean score difference between them was 0.0, indicating a negative intracutaneous reactivity response. Based on these results, the Mg alloy extract was negative for intracutaneous reactivity under the test conditions.

**Fig. 4:**
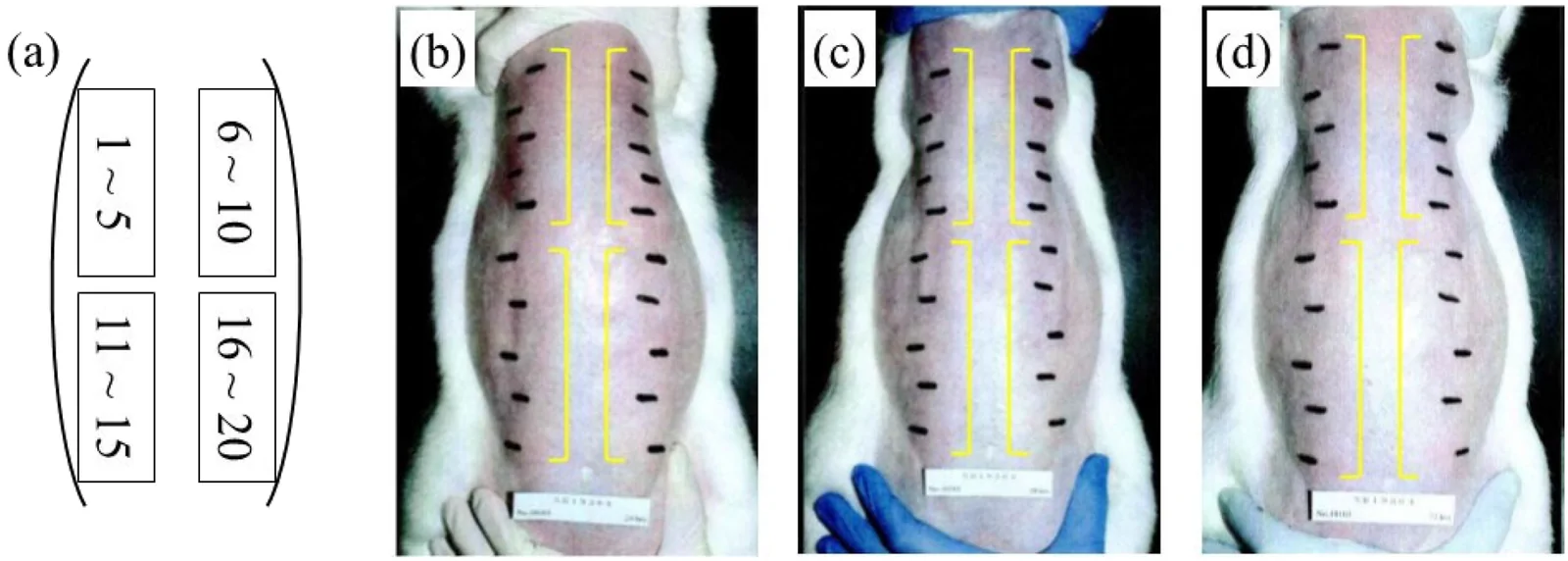
Appearances of Mg alloy extracts-injected rabbits: (a) Injection sites of polar extracts (1–5) and blank controls (6–10), non-polar extracts (11–15) and blank control (16–20), (b) 24 h, (c) 48 h, and (d) 72 h after injection.

### 3.3 In vivo study

The short-term degradation behavior of Mg alloy HC screws implanted into the lateral distal femoral condyle of rabbits was evaluated at 3, 6, and 9 weeks post-implantation using radiological and histological analyses. Fig. 5 shows representative radiographs of the distal femoral condyles obtained immediately after harvesting. Although inter-individual variability was observed, radiolucent areas consistent with gas cavities were clearly visible around the screws at 3 weeks postoperatively. These radiolucent areas gradually decreased over time and were scarcely detectable at 9 weeks. At 9 weeks, the screw became radiographically indistinct, and cortical bone bridging over the trailing end of the screw was observed. Fig. 6 shows representative three-dimensional micro-CT images and the corresponding relative volumes of the Mg alloy HC screws at each implantation time point. The Mg alloy HC screws gradually thinned as degradation progressed over time. The residual screw volume fraction (relative to initial volume) decreased to approximately 50% by 6 weeks postoperatively and further decreased to 44% at 9 weeks.

**Fig. 5:**
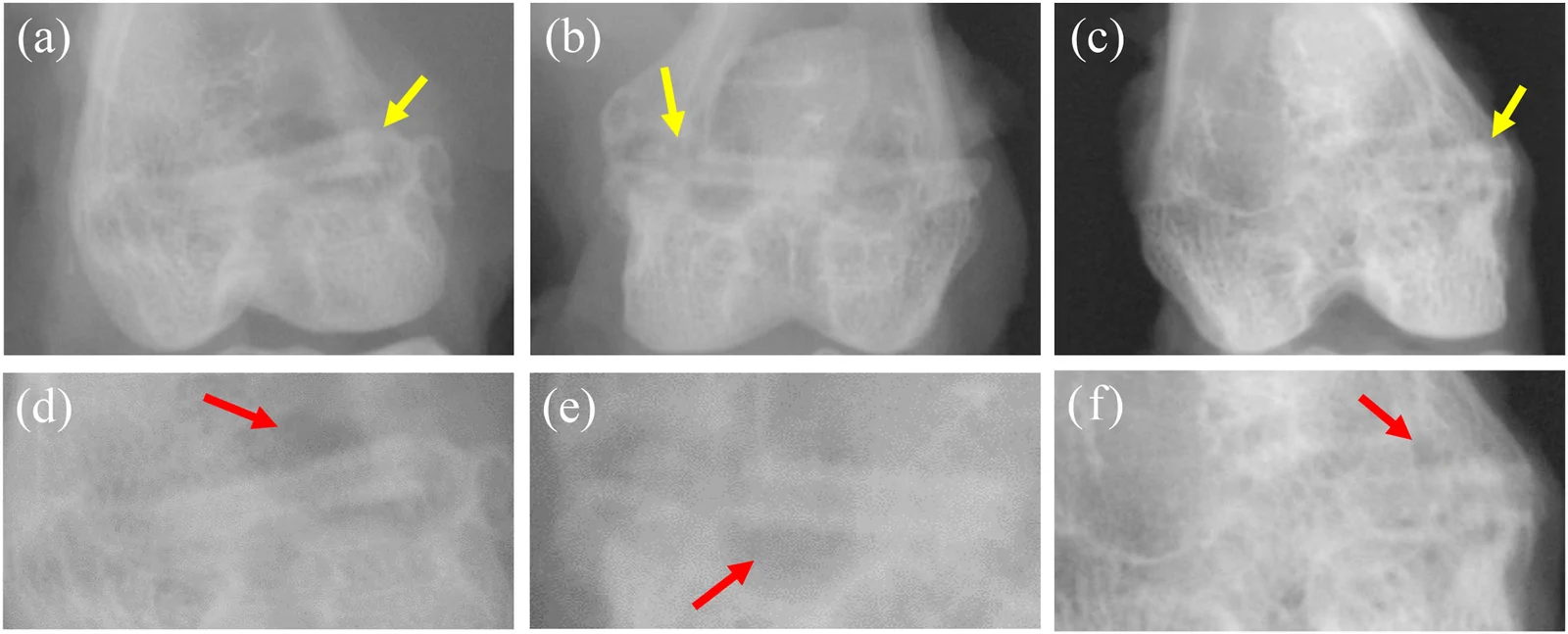
Representative radiographs of the distal femoral condyles at 3 weeks (a, d), 6 weeks (b, e), and 9 weeks (c, f) post-implantation. (d–f) are higher-magnification images of (a–c), respectively. The yellow arrows indicate the implanted screws, and the red arrows indicate radiolucent areas around the screws.

**Fig. 6:**
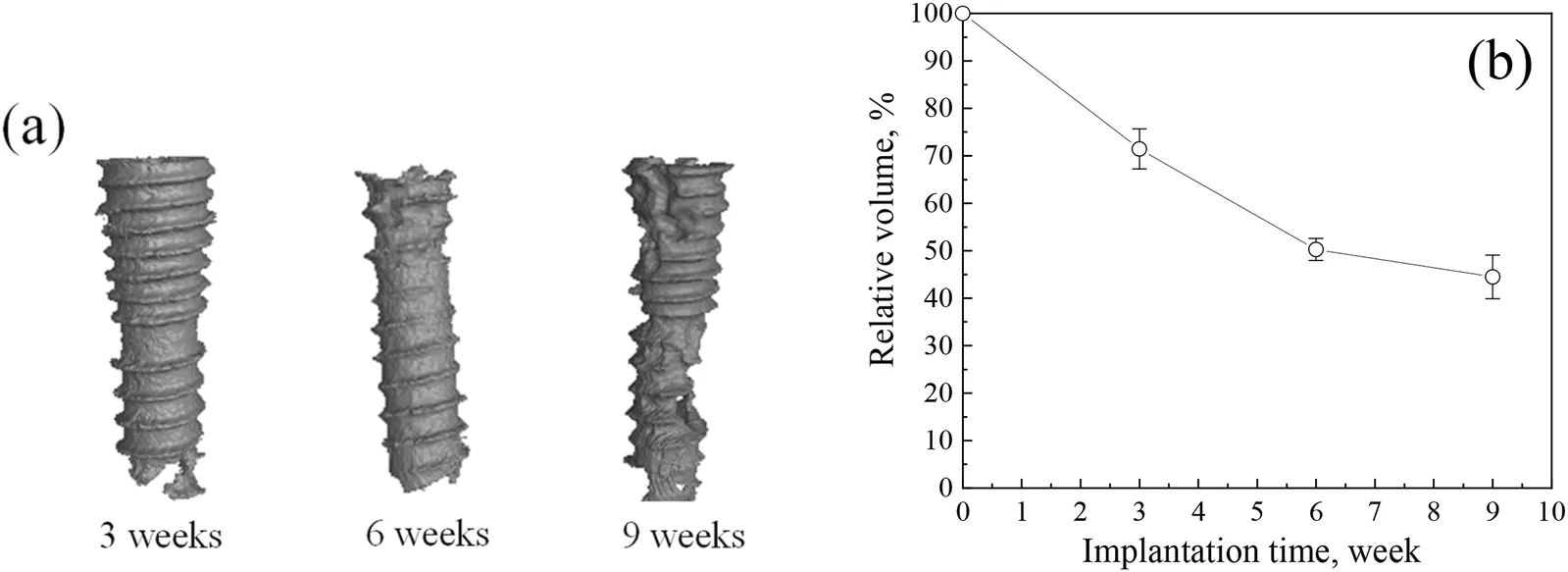
Representative three-dimensional micro-CT images of the Mg alloy HC screws at 3, 6, and 9 weeks post-implantation (a) and changes in relative volume over time (b).

TB-stained histological images of harvested distal femoral condyle samples are shown in Fig. 7. Degradation of the implanted Mg alloy HC screws was initiated at the surface and progressed over time. Partial screw resorption was evident at 9 weeks postoperatively. After 6 weeks, new bone formation and direct bone-screw contact were observed at the screw-bone interface, suggesting ongoing bone remodeling activity. In addition, gas cavity formation was observed over the first 3 weeks postoperatively, as suggested by the radiographic findings (Fig. 5). These gas cavities reduced the cancellous bone tissue around the leading end of the screw, resulting in bone–screw contact being primarily limited to the cortical bone, which is a characteristic feature commonly observed during the degradation of Mg-based implants. However, as shown in Fig. 8, the gas cavities gradually decreased over time, accompanied by progressive new bone formation, suggesting that the degradation process did not adversely affect the surrounding tissue response. For these histological specimens, inflammatory response, osteocyte necrosis, fibrous tissue-screw contact, and bone tissue-screw contact were evaluated using a semi-quantitative scoring system (0–5), and the results are shown in Table 3. No inflammatory response or osteocyte necrosis was observed around the screws throughout the implantation period (Score = 0). The fibrous tissue-screw contact score decreased from 2.5 at 3 weeks to 2.0 at 9 weeks, whereas the bone-screw contact score increased from 0.5 at 3 weeks to 2.7 at 9 weeks.

**Fig. 7:**
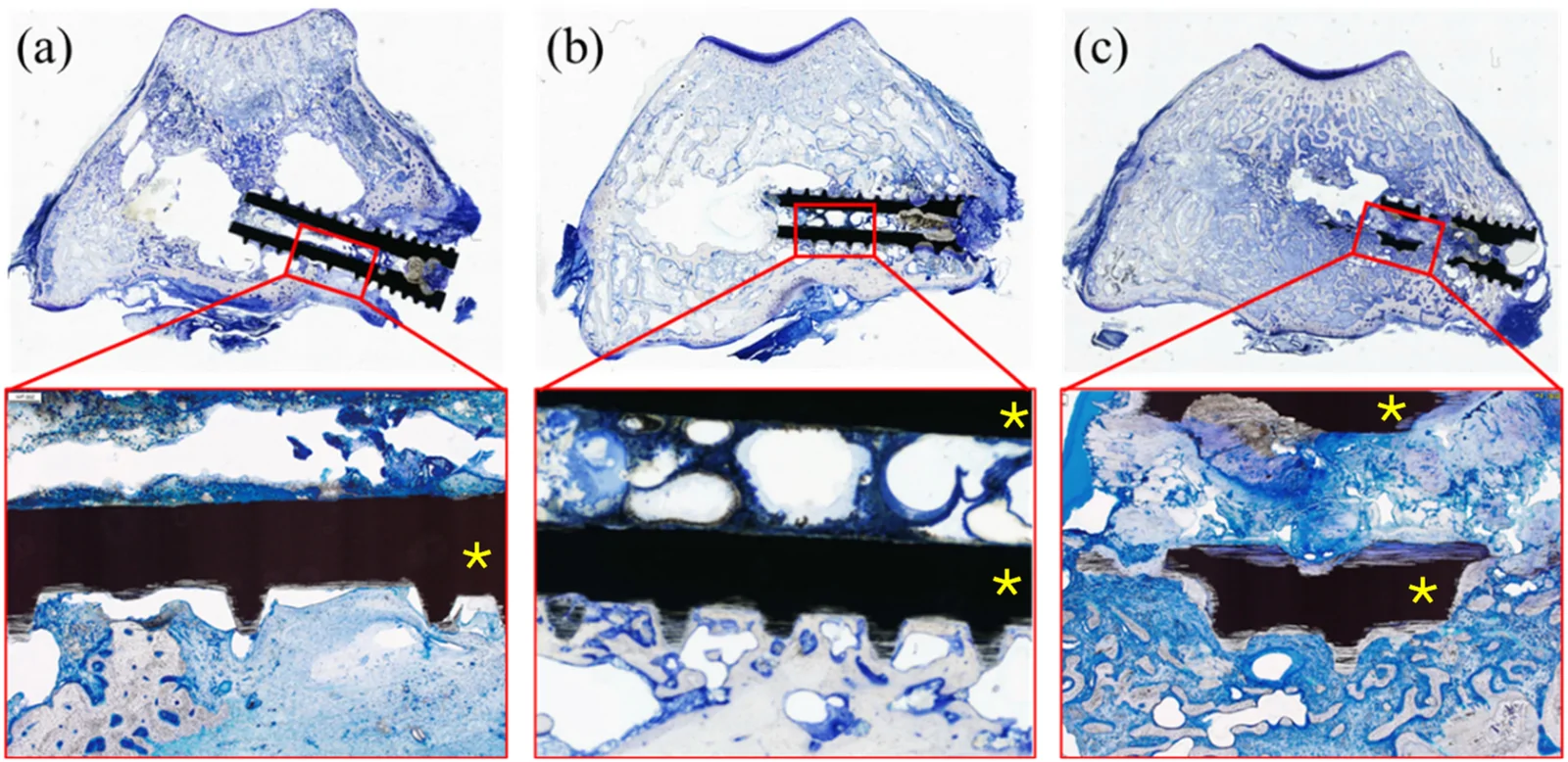
TB-stained histological images of distal femoral condyle samples harvested at 3 weeks (a), 6 weeks (b), and 9 weeks (c) post-implantation. The yellow asterisks indicate the implanted screw body.

**Fig. 8:**
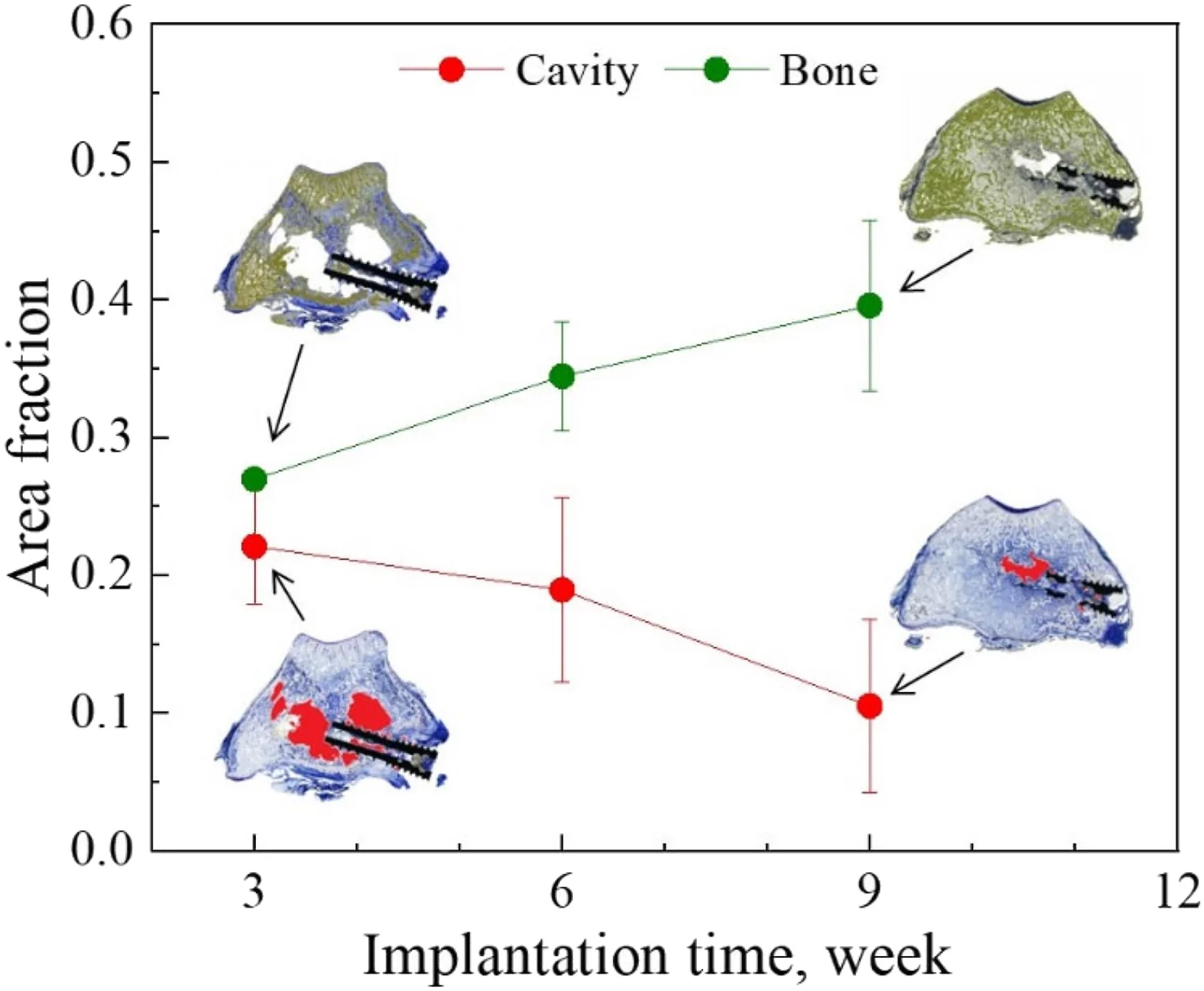
Changes in the area fractions of gas cavities and bone tissue relative to the total cross-section of the distal femoral condyle sample.

**Table 3:** Semi-quantitative scoring values of biological response for the implanted Mg alloy HC screw.

Implantation time	Mean score^a^			
	Inflammation	Osteocyte necrosis	Fibrous tissue-implant contact	Bone-implant contact
3 weeks	0.0	0.0	2.5 ± 0.7	0.5 ± 0.7
6 weeks	0.0	0.0	2.0 ± 0.0	2.0 ± 1.0
9 weeks	0.0	0.0	2.0 ± 1.0	2.7 ± 0.6

a Grading of findings: no change 0, minimal 1, mild 2, moderate 3, marked 4, and severe/extreme 5.

To investigate the long-term biodegradation behavior of the Mg alloy HC screws, histological and elemental analyses were performed on femoral specimens harvested 10 months after implantation. As shown in Fig. 9(a), no adverse findings—such as inflammation, gas cavities, necrosis, or impaired bone formation—were observed at the implantation site or in the surrounding tissues. Elemental mapping of Calcium (Ca) and Mg demonstrated that most of the implanted screw had degraded and been resorbed. Furthermore, a substantial amount of newly formed bone tissue was observed at the original implantation site and around the screw residue, indicating that the screw was osteoconductive (Fig. 9(b)).

**Fig. 9:**
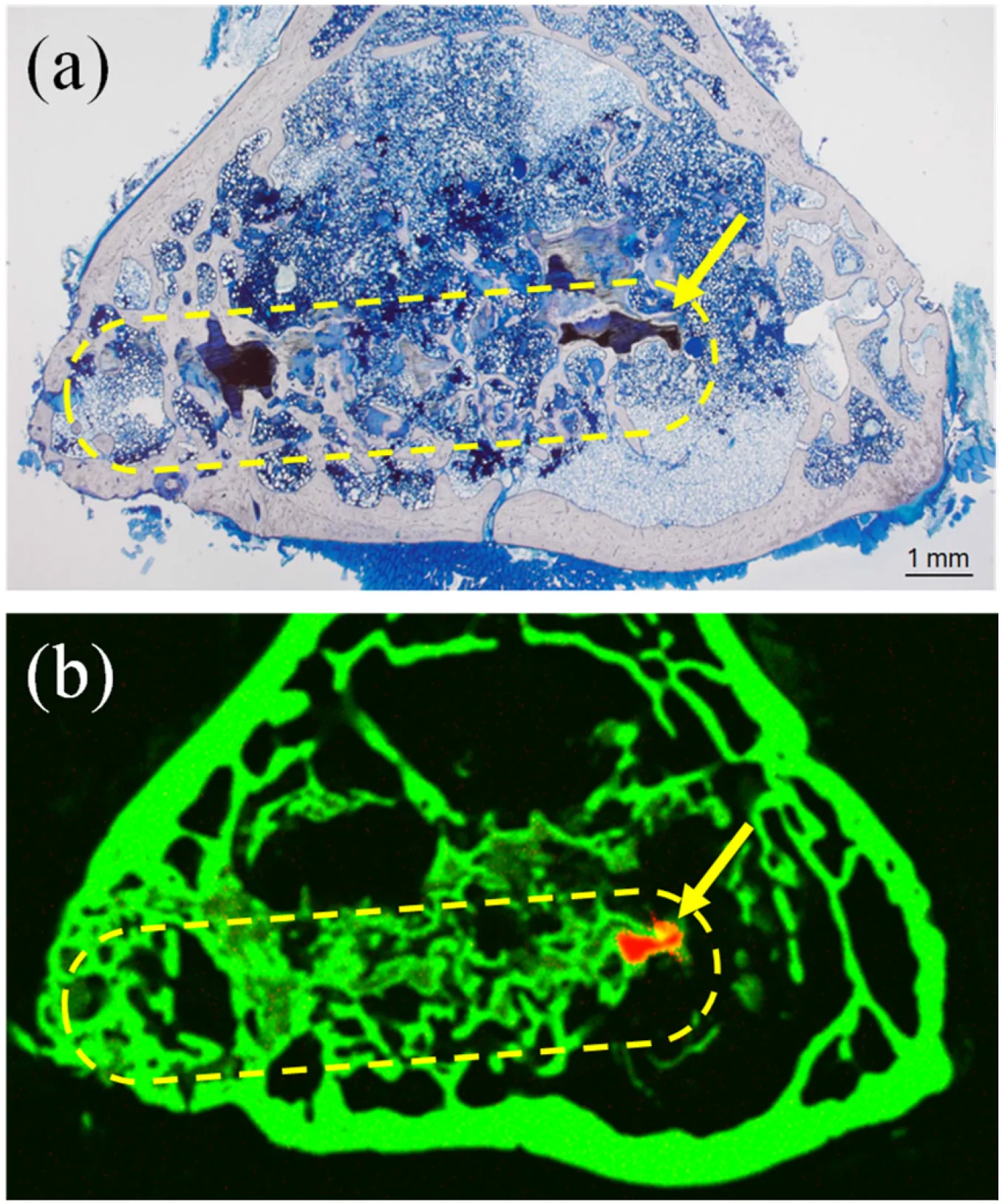
Distal femoral condyle sample harvested at 10 months post-implantation of the developed Mg alloy HC screw: TB-stained histological specimen image (a) and elemental mapping image (b) of Ca (green) and Mg (red) by XRF. The yellow dotted circle and arrow indicate the screw implantation site and the almost degraded Mg alloy screw, respectively.

To investigate the biodistribution of alloying elements derived from degraded and resorbed Mg alloy HC screws, quantitative analysis was performed by ICP-MS in the blood and target organs of rabbits at 9 weeks and 10 months post-implantation. Table 4 shows the concentrations of these elements in the blood and target organs. No significant differences were observed in the concentrations of Mg, Zn, Mn, and Zr in the blood or any organ between the 9-week and 10-month time points. For the rare-earth elements Nd and Y, concentrations in the blood and kidneys were comparable between the two time points, whereas a slight increase was observed in the liver and spleen at 10 months post-implantation.

**Table 4:** Quantities of alloying elements in the blood and target organs of rabbits harvested at 9 weeks and 10 months post-implantations.

Sample	Implantation time	Alloying element, μg/g					
		Mg	Nd	Y	Zn	Mn	Zr
Blood	9 weeks	67 ± 5	L.Q.	L.Q.	4.1 ± 0.1	0.019 ± 0.002	L.Q.
	10 months	50 ± 1	0.016 ± 0.001	L.Q.	3.4 ± 0.3	0.039 ± 0.001	L.Q.

Liver	9 weeks	180 ± 3	L.Q.	L.Q.	26 ± 1	1.6 ± 0.1	L.Q.
	10 months	120 ± 4	0.31 ± 0.024	0.04 ± 0.003	22 ± 1	0.71 ± 0.04	L.Q.

Kidney	9 weeks	210 ± 7	L.Q.	L.Q.	20 ± 4	1.7 ± 0.3	L.Q.
	10 months	170 ± 9	0.011 ± 0.002	0.012 ± 0.002	21 ± 1	1.1 ± 0.2	0.011 ± 0.001

Spleen	9 weeks	140 ± 2	L.Q.	L.Q.	13 ± 2	0.23 ± 0.001	L.Q.
	10 months	180 ± 11	1.8 ± 0.01	0.43 ± 0.003	18 ± 2	0.25 ± 0.003	0.09 ± 0.001

L.Q.: Limit of quantitation.

### 3.4 Discussion

This study was designed as a preclinical evaluation of fully threaded FAsorbMg alloy HC screws toward clinical application, focusing on mechanical performance, biological safety, and in vivo degradation behavior rather than on fracture healing under load-bearing conditions. Although the rabbit distal femoral condyle model does not fully mimic clinical fracture fixation scenarios, it provides a well-established model for evaluating local tissue responses and implant degradation.

The Mg alloy HC screw tested in accordance with ASTM F543-17 demonstrated approximately twice the torsion torque of an already approved bioresorbable PLLA/HA screw. Furthermore, it exhibited insertion performance comparable to that of a Herbert-type Ti alloy screw and a push-out force approximately 1.4 times higher. The improved mechanical properties of the Mg alloy HC screw, including higher torsional strength and enhanced fixation performance, may reduce the risk of early loosening and displacement at the fracture site, which are critical factors for successful bone healing. According to our review of previous studies on HC screws—most of which have investigated Ti alloy screws—the full-threaded type has exhibited superior mechanical performance compared with the partially threaded type in screw design [[29], [30], [31], [32],^34^,^35^]. Furthermore, for the same screw design, no significant difference in mechanical performance has been reported between Mg alloy and Ti alloy screws [36,37]. The mechanical testing results of our Mg alloy HC screw are consistent with these previous findings and demonstrate clinically acceptable and sufficient initial mechanical safety. Considering that Mg alloy screws are progressively degraded and resorbed into the body, a fully threaded design that provides higher bone fixation performance during the initial implantation period is reasonable. Furthermore, Jia et al. reported that fully threaded Ti alloy HC screws demonstrate a significant clinical advantage over partially threaded ones in the treatment of femoral neck fractures [34], suggesting that similar clinical advantages may be expected for fully threaded Mg alloy HC screws.

The biological safety of the FAsorbMg alloy used as the screw material in this study was evaluated through GLP-compliant testing, demonstrating no evidence of cytotoxicity, skin sensitization, or intracutaneous reactivity. Although potential safety concerns have been raised about the alloying elements, particularly rare-earth elements, the results of these biological safety assessments indicate a favorable biological profile for the alloy. Therefore, the FAsorbMg alloy is considered to possess a safety profile comparable to or superior to that of previously reported clinically applied Mg materials, such as WE43 alloys and high-purity Mg [25].

The degradation behavior of the Mg alloy HC screw within bone tissue, together with the associated gas cavity formation and bone formation, was investigated in vivo using a rabbit femoral condyle model. After implantation, degradation of the screw progressed gradually from the surface, and gas cavities were observed around the screw at 3 weeks post-implantation. The gas cavity formation led to a temporary reduction in bone-screw contact within the cancellous bone, restricting contact primarily to the cortical bone, therefore raising concerns about potential peri-screw bone weakening and reduced fixation strength. However, after 3 weeks, the gas cavities gradually decreased, while new bone formation increased, accompanied by direct contact of bone tissue with the screw surface and evidence of bone remodeling. As a result, the bone volume fraction increased, and favorable osseointegration with the screw was progressively established over time, indicating that the initially anticipated risks were transient. Furthermore, at 10 months post-implantation, the screw was almost completely degraded and resorbed, and the gas cavities had completely disappeared. In this study, hydrogen gas evolution and the resulting gas cavity formation, a well-known key concern for Mg-based implants, were transient and did not impair bone formation or implant stability. Furthermore, these findings are consistent with previous reports indicating that gas cavity formation is self-limiting and clinically manageable [[7], [8], [9], [10], [11]]. Furthermore, as reported in previous studies [38,39], a high-density trabecular bone structure was observed at the screw implantation site, which may be attributed to the osteogenic effects of Mg ions released during screw degradation. Throughout the process from screw degradation to resorption, there were no adverse histological findings, and the results were as we expected.

On the other hand, previous studies on metallic implants have reported that their degradation products may accumulate in target organs such as the liver, kidney, spleen, and lymph nodes [40]. Rössig et al. investigated the effects of a MgLiAlRE (LAE442) alloy intramedullary nail system implanted in the tibiae of sheep for 24 weeks and reported that rare-earth elements contained in the alloy—cerium, lanthanum, neodymium, and praseodymium—were detected in the liver, kidney, and spleen; however, no evidence of inflammation, necrosis, or other pathological changes was observed in these organs [41]. Angrisani et al. examined the long-term effects of LAE442 alloy pins implanted into the rabbit tibial medullary cavity over 3.5 years. They reported that, although rare-earth elements accumulated in the target organs, no physiological or toxicological effects were observed. Additionally, they suggested that immune cells (macrophages) might be involved in the migration and accumulation of rare-earth elements to target organs [42]. Similarly, our in vivo results showed slight accumulation of the alloy-derived rare-earth elements neodymium and yttrium in the liver and spleen of rabbits at 10 months post-implantation, and no significant pathological changes were observed in any target organ at necropsy. These findings suggest that the systemic effects of the rare-earth elements released from the FAsorbMg alloy on target organs are likely minimal.

Based on the above findings, the fully threaded FAsorbMg alloy HC screw developed in this study can be considered to identify no safety concerns for clinical use. However, this study has several limitations. First, a fracture model under load-bearing conditions was not employed; therefore, the direct clinical performance in fracture fixation has not been validated. Second, the sample size was limited, and the effect of sample size on degradation behaviors and biological responses has not been examined. Future studies should evaluate the performance of Mg alloy HC screws with various sizes under clinically relevant fracture models and load-bearing conditions to further validate their translational potential. Furthermore, from an orthopedic surgeon's perspective, the following clinically relevant issues should be systematically addressed in future clinical studies of the Mg alloy HC screws: (1) indications and contraindications; (2) the risk of screw loosening and subsequent displacement at the fracture site; (3) postoperative management strategies, including the need for external fixation and specific considerations for rehabilitation; and (4) appropriate postoperative radiographic assessment methods.

## 4 Conclusions

In this study, we assessed the mechanical and biological safety and in vivo biodegradation behavior of the full-thread FAsorbMg alloy HC screw to evaluate its safety for clinical use. From both mechanical and biological perspectives, the full-thread Mg alloy HC screw exhibited excellent insertion and bone fixation performance, comparable to or superior to that of clinically approved HC screws. In addition, the screw showed excellent biocompatibility, and its overall performance was considered clinically acceptable. In vivo studies revealed that degradation progressed gradually from the surface over time, and the screw was almost completely degraded and resorbed by 10 months post-implantation. During this process, gas cavity formation and subsequent reduction around the screw, as well as bone formation and remodeling, were observed; however, these changes remained within clinically manageable limits. Furthermore, the effect of screw-derived degradation products on target organs appears to be minimal. In conclusion, the fully threaded Mg alloy HC screw developed in this study demonstrated favorable mechanical properties, biocompatibility, and in vivo degradation in a preclinical model. These findings suggest that the developed Mg alloy screw could be an alternative to conventional implants, potentially eliminating the need for secondary removal surgery.

## Author's contributions

Noguchi H: Writing-Original Draft, Conceptualization, Methodology, Investigation, Data curation, Formal analysis, Visualization, Validation. Hanada K: Writing-Original Draft, Conceptualization, Investigation, Data curation, Formal analysis, Visualization, Validation. Watanabe R: Investigation. Totoki Y: Investigation. Tomaru Y: Investigation. Ikumi A: Investigation. Kitagawa A: Funding acquisition, Project Administration, Resources. Orui S: Data curation. Zhang L: Writing-Reviewing & Editing, Methodology, Mishima H: Supervision.

## Availability of data and materials

The data supporting the findings of this study are available from the corresponding author upon reasonable request.

## Declaration of generative AI in scientific writing

No generative AI or AI-assisted technologies were used in the preparation of this manuscript.

## Funding

This research was funded by the Japan Agency for Medical Research and Development (AMED) through the Medical Device Innovation Promotion Project (Grant No. JP20he1302028).

## Declaration of competing interest

The authors declare the following financial interests/personal relationships which may be considered as potential competing interests:Hiroshi Noguchi reports financial support was provided by the Japan Agency for Medical Research and Development (AMED). If there are other authors, they declare that they have no known competing financial interests or personal relationships that could have appeared to influence the work reported in this paper.
