# Supplementary material for: Preclinical evaluation of a fully threaded biodegradable magnesium alloy headless compression screw for enhanced fixation in small bones
Source: J Orthop Translat. 2026 Aug 27;60:101201. doi: 10.1016/j.jot.2026.101201 (PMC13662688; doi:10.1016/j.jot.2026.101201)
Supplement: Supplementary file 1 [file mmc1.docx]

**Supplementary material**

Table Raw data from the initial mechanical safety assessment of the developed Mg alloy HC screw and commercially available screws.

|  | Max. insertion torque /Nm | Yield torsion torque /Nm | Max. torsion torque /Nm | Max. push out force /N |
| --- | --- | --- | --- | --- |
| Mg alloy HC screw | 0.13533  0.15987  0.14646  0.13646  0.15021 | 0.17797  0.21251  0.19617  0.20373  0.19872 | 0.30479  0.33912  0.28573  0.33761  0.27533 | 202.8  230.5  202.6  208.4  218.3 |
| Ti alloy screw | 0.19325  0.18578  0.16305  0.16847  0.17081 | 0.37105  0.85891  0.44755  0.88491  0.89162  0.93111 | 0.46065  1.24437  1.70675  1.29922  1.30423  1.30484 | 174.4  158.6  157  140.4  146.3 |
| PLLA/HA screw | 0.08675  0.0892  0.08441  0.12605  0.06499  0.0885 | 0.09963  0.09528  0.09899  0.10099  0.09653 | 0.14018  0.1374  0.14751  0.14196  0.14245 | 20.4  46.8  40.8  48.8  40.7  29.8 |
